# Development of an integrated model of care for allogeneic stem cell transplantation facilitated by eHealth—the SMILe study

**DOI:** 10.1007/s00520-021-06328-0

**Published:** 2021-07-05

**Authors:** Lynn Leppla, Anja Schmid, Sabine Valenta, Juliane Mielke, Sonja Beckmann, Janette Ribaut, Alexandra Teynor, Fabienne Dobbels, Nathalie Duerinckx, Robert Zeiser, Monika Engelhardt, Sabine Gerull, Sabina De Geest, the SMILe study team, Dora Bolliger, Yves Chalandon, Sabina De DGeest, Sabine Degen, Fabienne Dobbels, Nathalie Duerinckx, Monika Engelhardt, Margerita Fürmann, Sabine Gerull, Florian Grossmann, Monika Hasemann, Philipp Heidegger, Anja Hermann, Sandra Hobelsberger, Mylen Husel, Katharina Koehly, Marina Lemcke, Lynn Leppla, Birgit Maier, Anne-Claire Mamez, Stavoula Masouridi, Juliane Mielke, Gyathri Nair, Daniela Neupert, Jakob Passweg, Stefan Pschenitza, Sigrun Reitwiessner, Jannette Ribaut, Dennis Rockstein, Urs Schanz, Anja Schmid, Helen Schoemans, Tobias Schulz, Vanessa Schumacher, Yulia Senft, Alexandra Teynor, Sabine Valenta, Viktor Werlitz, Verena Witzig-Brändli, Robert Zeiser

**Affiliations:** 1 Institute of Nursing Science, Department Public Health, University of Basel, Basel, Switzerland; 2 Department of Medicine I, Faculty of Medicine, Medical Center University of Freiburg, Freiburg im Breisgau, Germany; 3 Department of Hematology, University Hospital Basel, Basel, Switzerland; 4 Center of Clinical Nursing Science, University Hospital Zurich, Zürich, Switzerland; 5 Faculty of Computer Science, University of Applied Sciences Augsburg, Augsburg, Germany; 6 Academic Centre for Nursing and Midwifery, Department of Public Health and Primary Care, KU Leuven, Leuven, Belgium; 7 Nursing Science (INS), Department Public Health (DPH), Faculty of Medicine, University of Basel, Bernoullistrasse 28, CH-4056 Basel, Switzerland

**Keywords:** Allogeneic stem cell transplantation, eHealth, Integrated care, Implementation science, Behavioral science, User-centered design, Agile software development, Intervention development

## Abstract

**Purpose:**

Allogeneic stem cell transplantation would benefit from re-engineering care towards an integrated eHealth-facilitated care model. With this paper we aim to: **(**1) describe the development of an integrated care model (ICM) in allogeneic **S**te**M**-cell-transplantat**I**on faci**L**itated by **e**Health (SMILe) by combining implementation, behavioral, and computer science methods (e.g., contextual analysis, Behavior Change Wheel, and user-centered design combined with agile software development); and (2) describe that model’s characteristics and its application in clinical practice.

**Methods:**

The SMILe intervention’s development consisted of four steps, with implementation science methods informing each: (1) planning its set-up within a theoretical foundation; (2) using behavioral science methods to develop the content; (3) choosing and developing its delivery method (human/technology) using behavioral and computer science methods; and (4) describing its characteristics and application in clinical practice.

**Results:**

The SMILe intervention is embedded within the eHealth enhanced Chronic Care Model, entailing four self-management intervention modules, targeting monitoring and follow-up of important medical and symptom-related parameters, infection prevention, medication adherence, and physical activity. Interventions are delivered partly face-to-face by a care coordinator embedded within the transplant team, and partly via the SMILeApp that connects patients to the transplant team, who can monitor and rapidly respond to any relevant changes within 1 year post-transplant.

**Conclusion:**

This paper provides stepwise guidance on how implementation, behavioral, and computer science methods can be used to develop interventions aiming to improve care for stem cell transplant patients in real-world clinical settings. This new care model is currently being tested in a hybrid I effectiveness-implementation trial.

**Supplementary Information:**

The online version contains supplementary material available at 10.1007/s00520-021-06328-0.

## Introduction

Although allogeneic stem cell transplantation (alloSCT) recipients’ survival has improved over recent years, significant risks remain for short- and long-term complications such as infections or graft-versus-host disease (GvHD) [1, 2]. Moreover, non-adherence to their therapeutic regimens is common and can negatively affect long-term outcomes [3–5]. Including psychosocial issues, alloSCT patients’ comprehensive care needs demand self-management interventions embedded in an integrated care model (ICM) [6]. Based on chronic illness management principles, using multidisciplinary team-based approaches spanning settings and care levels [7], ICMs strengthen person-centered care, potentially improving medical, behavioral, and economic outcomes [8].

One excellent example is the Chronic Care Model (CCM). Its four inner dimensions—self-management support, decision support, clinical information systems, and delivery system design—guide the re-engineering of acute-care-oriented models towards chronic care principles [9]. The more are combined, the stronger the effect [10]. Considering healthcare’s increasing digitalization, the updated eHealth enhanced Chronic Care Model (eCCM) explains how to strengthen all four dimensions via digitalization [11]. Systematic reviews and meta-analyses indicate that eHealth-facilitated ICMs improve biomedical, behavioral, psychosocial [12–14], and economic outcomes [13, 15]. In organ transplant recipients, these include improved medication adherence [16] and reduced re-hospitalizations [17]. In cancer patients, eHealth-facilitated care models integrating two or more eCCM dimensions led to, e.g., reduced symptom burden [18], re-hospitalizations, improved survival, quality of life [19, 20], and physical activity [21].

Regarding SCT care models, the only two RCTs focused respectively on one and two eCCM dimensions. For the first, Bryant et al. [22] implemented electronic patient-reported outcomes into routine care during the first 2 weeks post-SCT (n = 45 autologous; n = 31 allogeneic), followed by tailored self-management support leading to reduced peak symptom burden (*p* = 0.03). In the second, Syrjala et al. [23] found that for survivors > 3 years post-SCT (n = 182 autologous; n = 566 allogeneic), online self-management and decision support led to reduced treatment distress (*p* = 0.032). However, both trials focused on very specific treatment phases; and neither was conceptually embedded in an ICM or comprehensively addressed multiple eCCM dimensions.

Moreover, implementing eHealth-facilitated ICMs into routine care is often problematic [24]. Adoption and sustainment problems commonly prevent eHealth applications’ integration in established care models, with 44 to 67% of patients discontinuing the use [25–27]. Explanations include poor fit to context-dependent variables, deficits regarding behavioral effectiveness, and problems with the technology not meeting users’ needs [28].

Combining implementation (e.g., contextual analysis), behavioral (e.g., behavior change theories), and computer science methods (e.g., agile software development, user-centered design) to develop eHealth-facilitated interventions could solve such problems [24, 28, 29]. Implementation science sets the long-range goal—sustainably improving the quality and effectiveness of patient care [30]. This means integrating methodological considerations such as stakeholder involvement, contextual analysis, and the choice and application of context-adapted implementation strategies [31]. Combined with a theory-guided content development using behavioral science and by developing the necessary technology around end-user needs and preferences using agile software development processes, maximize its usability and accessibility [28, 29, 32]. For eHealth component production, this combination promises fast, iteratively improved software versions that can be discussed regularly with end users (patients, clinicians) [33]. Following this formula, implementation/behavioral/computer science fusions should facilitate user-friendly, contextually targeted eHealth components ready to be embedded within ICMs.

Although alloSCT patients can clearly benefit from an eHealth-facilitated ICM, no such model currently exists nor has been prepared for implementation in real-world settings. Therefore, we are developing, implementing, and testing an allogeneic **S**te**M**-cell-transplantat**I**on faci**L**itated by **e**Health **I**ntegrated **C**are **M**odel (SMILe-ICM) combining implementation and behavioral methods with computer science methods. This article first reports on the methods used to develop the SMILe-ICM, then describes its characteristics and application in clinical practice.

## Methods and results

**Fig. 1:**
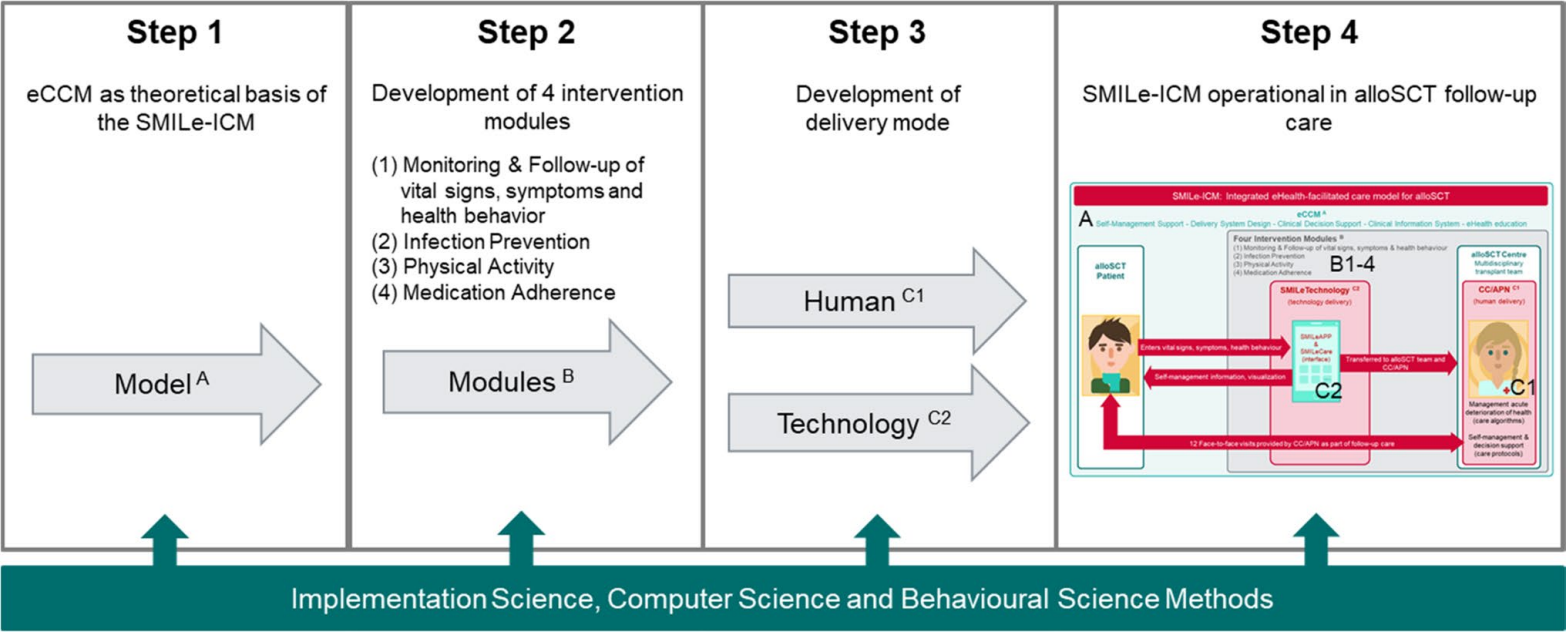
The three subsequent steps of the development process building up to the SMILe-ICM described in step 4. *Note:* A, B, C also refer to Fig. 2 were the same elements can be found within the visualization of the SMILe integrated care model (ICM)

The development phase consisted of a sequence of four steps, with implementation science methods informing each (Fig. 1): (1) Choose the SMILe-ICM’s theoretical foundation; (2) Develop four theory-guided self-management intervention modules; and (3) Choose and develop the intervention’s delivery method(s), i.e., human and/or technology. With the SMILe-ICM’s three-step development complete, the fourth step is to report on those steps, along with its characteristics and its application in daily clinical practice. All four steps are described below.

**Fig. 2:**
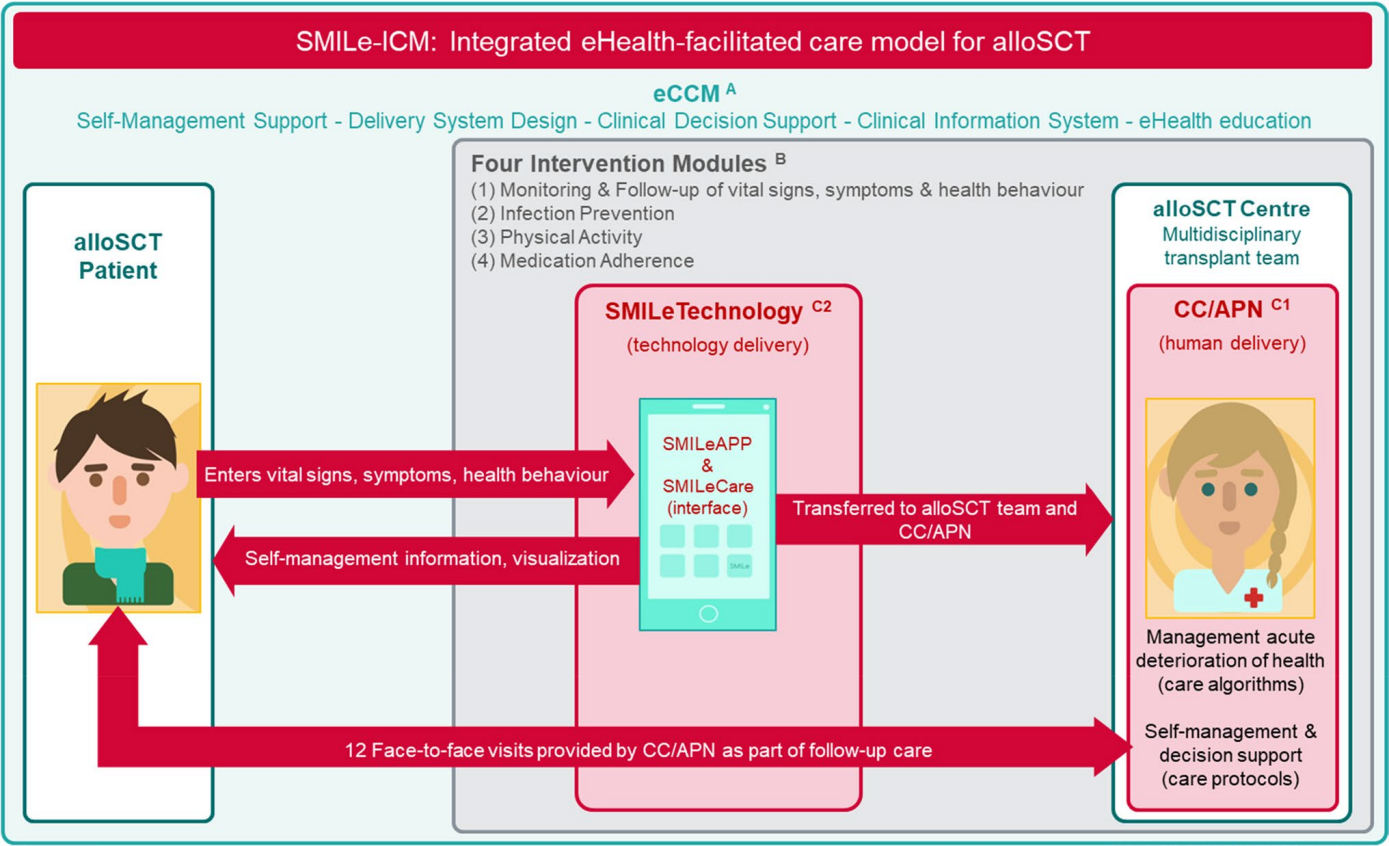
The SMILe integrated care model. *Note: A* = *Five dimensions of the eHealth enhanced Chronic Care Model, B* = *Four intervention modules delivered partly *via* human (alloSCT Transplant Team and CC* = *C1) and partly *via* technology components (*= *C2); CC*, *care coordinator;* *APN*, *advanced practice nurse*

- Step1: The SMILe-ICM’s theoretical foundation
  The SMILe-ICM is grounded in the eCCM (Fig. 1 step 1; Fig. 2 A), a choice based on our contextual analysis, findings can be found in detail elsewhere [34]. Data from two surveys (60 patients/5 clinicians), three clinician focus groups and ten patient interviews indicated that the existing care model is mainly acute care-driven: it focuses on diagnosing and curing patients; inpatient and outpatient care are separate, with limited collaboration between their clinician teams; outpatient alloSCT follow-up is primarily physician-centered, focusing mainly on medical aspects; no nurses are involved in care delivery and little attention is devoted to self-management support. Use of eCCM dimensions would quickly allow connections between inpatient and outpatient care teams by encouraging interdisciplinary teamwork. Furthermore, far from replacing human contact between patients and providers, eHealth should supplement that contact, helping maintain continuity of care and self-management support.
- Step: 2 Theory-guided content development of the intervention modules
  Our contextual analysis, empirical evidence, and clinicians’ and patients’ endorsements led us to produce four self-management intervention modules: monitoring and follow-up, infection prevention, medication adherence, and physical activity (Fig. 2 B). Content development followed the Behavior-Change Wheel (BCW). A widely used amalgamation of 19 behavior change theories [35], the BCW helps its users understand, explain, and change behaviors via stepwise development processes. With the Capability-Opportunity-Motivation-Behavior (COM-B) Model at its hub, the BCW meshes well with the Theoretical Domains Framework (TDF) [35], which includes 14 domains, synthesizing key theoretical constructs used in behavioral theories such as knowledge, skills, goals, or beliefs relating to the COM-B component.
  For developing our intervention modules’ content, we established an interdisciplinary team with expertise in implementation and behavioral science methods (nursing scientists, psychologist). Pairs of researchers conducted a literature search to identify empirical evidence regarding a specific self-management issue, its determinants, and possible target behaviors. Determinants were derived from empirical evidence, the contextual analysis [34], and clinical expertise of the team into the COM-B taxonomy, then discussed by the entire group. Where teams found multiple behaviors, the group chose which to target.
  After choosing intervention functions for all targets, we selected appropriate TDF domains, behavioral change techniques (BCTs), and applied APEASE criteria (affordability, practicability, effectiveness, acceptability, safety, and equity) to each. Finally, the teams carefully reflected upon the mode of delivery (face-to-face and/or technology-based) and added an additional step not covered by the BCW. We wrote comprehensive protocols for the intervention segments’ face-to-face visits and full descriptions of the functionalities to be digitized as user stories [36, 37].
  User stories are commonly formulated in a role-feature-reason format [38]. Each software functionality is presented as a sentence specifying the target *user/role,* the desired *feature*, and a reason/*expected outcome*, e.g., “As a *patient/clinician,* I want to *monitor my pain intensity* so that I can *keep track of my pain trajectory*.” By supporting the translation of BCTs into software features, user stories inform the iterative software development process. Once the stories were articulated, they were transferred to the software development team, starting the digitalization process [36].

### Description of the four intervention modules

To underpin our intervention modules’ content, seven intervention functions reflecting all TDF domains and 42 different BCTs from Michie’s taxonomy were chosen [35]. These informed 39 user stories. The following paragraphs describe the intervention modules (Fig. 2 B). Additional details for all modules regarding the target behaviors, behavioral issues, intervention components, user stories, behavior change techniques, intervention functions, TDF domains, and COM-B dimensions are presented in Supplementary Table 1.

#### Monitoring and follow-up

This module targets alloSCT patients’ insecurity regarding recognizing and reacting to new symptoms [34]. Our literature review and feedback cycles indicated that remote monitoring of patient-reported data effectively allows both accurate symptom recognition and timely reactions [18]. To support this behavior, we composed an initial set of 17 parameters for daily monitoring.

Parameter relevance was decided via an online survey of 12 German-speaking alloSCT experts. Using 6-point Likert-type scales (0 = not at all relevant – 5 = extremely relevant), these experts were asked to rate the importance of symptoms covered by the PROVIVO alloSCT patient-reported outcome questionnaire [39]. Inclusion of those with median ratings ≥ 4 led to a selection of 12 from the original PROVIVO questionnaire, plus one more recommended by the group: signs of bleeding. The experts also added temperature, blood pressure, weight, and general wellbeing, resulting in 17 items to be monitored for this module. To support appropriate reactions, we collaborated closely with physicians involved in alloSCT care to define meaningful cut-off levels and feedback algorithms for each parameter (e.g., temperature > 38.5: contact the center immediately). Table 1 exemplarily summarizes the intervention modules’ mechanisms for behavior change towards adequately recognizing, evaluating, and reacting to new symptoms.

**Table 1:** Description of the SMILe-ICM content and mechanism of change using the BCW at the example of the monitoring and follow-up module

Module: Monitoring and follow-up Table 1							
Target behavior	Problem based on context analysis and evidence	Content to tackle the problem			Mechanism of change		
		Mode of delivery Human and technology components	User-story (As a…I want…so that)	BCTs	Functions	COM-B	TDF
Improved recognition, evaluation, and acting upon symptom	• Uncertainty about symptom assessment	• CC explains and trains symptom assessment and SMILeApp use • CC evaluates SMILeApp use with patients and care-givers during visits • SMILeApp supports symptom assessment • SMILeCare facilitates symptom monitoring by CC and praises patients in visits	As a patient I want a system to assess symptoms so that I feel more secure As a CC I want a system to monitor symptoms of patients so that I can detect complications early	9.1 Credible source 4.1 Instruction perform the behavior 5.1 Info health consequences 6.1 Demonstration of the behavior 2.2 Feedback on behavior 2.7 Feedback outcome of behavior 8.1 Behavioral practice 15.1 Verbal persuasion 2.3 Self-Monitoring of behavior 12.5 Adding objects 2.5 Monitor outcome behavior by others without feedback 10.4 Social reward	Training Persuasion	Physical capability	S
	• Uncertainty about symptom judgement and reporting • High cognitive dysfunction and fatigue levels in alloSCT patients • Impaired retrospective recalling of symptoms • Improved survival, quality of life and lower re-admission rate through electronic symptom monitoring with self-management support	• CC explains critical symptoms and trains how to react if problems occur with patients and care-givers • CC discusses frequency of SMILeApp use by patients and if they managed to enter data as agreed upon • CC provides feedback about development of parameters and outcomes • SMILeApp provides feedback on severity of symptoms and how to act upon • SMILeApp provides a lexicon with self-management instructions	As a patient I want a daily reminder for using the system so that I do not forget to enter my data As a patient, I want a feedback on my self-assessed vital signs and symptoms so that I have support in my self-management and decision making As a patient, I want written information 24/7 available so that I can look up discussed information	9.1 Credible source 4.1 Instruction perform the behavior 5.1 Info health consequences 6.1 Demonstration of the behavior 8.1 Behavioral practice 7.1 Prompts/ cues 1.2 Problem solving 3.1 Social support 1.1 Goal setting 8.3 Habit formation 1.4 Action planning 1.5 Review goals (behavioral) 2.6 Biofeedback 2.7 Feedback outcome of behavior 5.1 Info health consequences	Education Training Enablement	Psychological capability	K MAD Br
Improved recognition, evaluation and acting upon symptoms	• Patients and clinicians would benefit from monitoring of critical symptoms, • No system available, • > 70% of patients would share their da or use a App from hospital • Remote monitoring of symptoms improves survival	• SMILeApp provides opportunity to monitor symptoms • CC encourages daily use of SMILeApp, identifies barriers to use it and set goals with patients and care-givers	As a patient I want to have the option to assess and share my entered data so that I have the certainty that someone is watching over me	12.5 Adding objects 1.2 Problem solving 1.1 Goal setting 1.4 Action planning	Training Enablement	Physical opportunity	Env
	• The knowledge that someone watches over your parameters gives a feeling of security, • Value of social support by peers and family, • Patients rate the importance of having technologies to share their data with others with a median of 8 (0–10)	• CC offers 12 face-to-face sessions over the first year post-alloSCT and cares for patients and families • SMILeCare connects patients virtually to CC and allows to overview incoming values • Patients observe other patients, family members using Apps for their health	As a CC, I want a system to monitor important parameters of patients at home within the hospital so that I can detect complications early	6.1 Demonstration of the behavior 12.5 Adding objects	Modeling Enablement	Social opportunity	Si
	• Clinicians assume that patients might be more anxious when assessing symptoms at a regular basis, • Might increase contacts to hospital, • Patients feel secured and watched over, • Would value a system tracking their parameters	• CC reviews together with patients the use of the SMILeApp in each face-to-face session • CC praises the use of the SMILeApp • CC teach patients to leave their smartphone next to their bed as a reminder to enter parameters • SMILeApp provides an overview about patent parameter development over time so that they can observe changes	As a CC, I want an overview how frequent patients entered their data into the system so that I can give feedback As a patient I want positive feedback when I use the system on a regular basis so that I keep motivated As a patient I want an overview about my entered data so that I can see changes over time and feel motivated to continue	2.2 Feedback on behavior 10.4 Social reward 7.1 Prompt and cues 8.3 Habit formation	Incentivization Training Enablement	Automatic motivation	Em
	• Patients are affright to get re-hospitalized, • Patients believe that monitoring of medical parameters is important • Early recognition decreases re-hospitalization, costs, and prevents comorbidities	• CC discusses with patients and care-givers that monitoring of symptoms can help to detect complications early and may improve long-term outcomes • CC offers patients and care-givers to call in terms of insecurity	As a patient, I want to have contact information within the system so that I know who to contact	5.3 Info social/environmental cons 5.1 Info health consequences	Education	Reflective motivation	B Cap O Id

*Note: BCT*, behavior change technique; *COM-B*, Capability, Opportunity Motivation Behavior; *TDF*, Theoretical Domains Framework; domains: *K*, knowledge; *S*, skills; *MAD*, memory attention, decision processes; *Br*, behavioral regulation; *Si*, social influences; *Env*, environmental context and resources; *Em*, emotion; *Int*, intension; *B cons*, beliefs about consequences; *B cap*, beliefs about capabilities; *O*, optimism; *G*, goals; *Id*, social/professional role and identity; *Reinf.*, reinforcement

#### Infection prevention

This module targets patients’ challenges regarding infection prevention measures, often leading to the significant burden of infection-related re-hospitalization—most common in the first 2 years post-alloSCT [2, 34]. Three target behaviors are covered: (1) adequate hand hygiene; (2) airborne pathogen-related risk reduction; and (3) safe food handling, preparation, and consumption. This module’s content required adaptivity, depending on participants’ time since transplantation and immunity status. Patients’ severely immuno-compromised or presenting signs of GvHD need stricter recommendations; for those with more stable immune systems, they can be loosened. This module also adds one monitoring parameter: *adherence to infection prevention measures*.

#### Medication adherence

This module responds to patients’ calls for immunosuppressant intake support [34]. More than 50% of alloSCT patients reported immunosuppressant non-adherence in view of errors in correct taking and timing [3–5]. Immunosuppressant medication non-adherence has been linked to GvHD, which frequently leads to poor clinical outcomes [3, 4, 40]. Therefore, the Medication Adherence module targets the implementation dimension of medication adherence (taking and timing behavior). This module also adds one monitoring parameter: *medication intake*. A detailed description of this module’s development process has been published elsewhere [37] and is a blueprint for how other modules were developed.

#### Physical activity

This module targets alloSCT patients’ commonly reduced physical capability due to pre-transplant treatment or post-transplant complications. In addition to lowering patients’ quality of life, these can shorten survival [41]. Conversely, improved physical condition is linked to improved health outcomes [34, 41]. Our contextual analysis indicated a need for support of physical activity before, during, and post-alloSCT.

However, review of all available evidence indicated that improving physical activity would be overly ambitious as a target behavior: patients are too weak in the first month’s post-alloSCT. Therefore, we reformulated the target behavior to “reducing sedentary bouts,” increasing patients’ physical activity alongside their energy levels. While we chose *daily step count* as an indirect measure of inactivity, this obviously also indicates physical activity. All patients receive step counters and training on how and when to wear them. This module adds *daily step count* as a monitoring parameter.

- Step: 3 Choice and development of intervention delivery methods

Both patients and clinicians preferred a combined face-to-face and eHealth-enhanced intervention [34]. We operationalized all four eCCM dimensions (Table 2) and determined the most effective delivery methods (human/technology).

**Table 2:** The eCCM dimensions and described operationalization of the SMILe-ICM

eCCM	SMILe technology	CC	Operationalization
SM-S		x	The CC provides patients with self-management support interventions beginning 2 weeks before until 1 year post-alloSCT, delivering 12 face-to-face sessions covering four modules
	x		Patients receive algorithm powered feedback based on entered parameters via the SMILeApp
	x	x	SMILeCare allows to detect complications early and allows to provide tailored additional face-to-face session
		x	In case of highly burdened patients, CC provides additional support and/or case-management
DSD	x		The use of information technology (SMILeApp and SMILeCare) is a new element and allows to adapt care-processes with the goal of optimizing both resource use and clinical outcomes
		x	Advanced practice oncology nurses need to be in place to work in the new role of a CC
		x	The introduction of the CC is a new element in alloSCT follow-up. Accordingly, care processes need to be adapted by the alloSCT center and weekly interdisciplinary discussion rounds should be implemented
	x	x	The SMILeApp contains and CC uses developed educational materials for each module
CDS	x		If serious symptoms are entered patients receive algorithm-based feedback how fast they should contact the transplant center
		x	The CC’s work is guided by protocols that build on the alloSCT centers’ clinical practice pattern guidelines and have been approved by the centers’ physicians
		x	The CC can discuss treatment decisions/changes pro-actively with attending physicians when necessary based on the monitoring
CIS	x		Vital signs, symptoms, and health behaviors of home dwelling alloSCT patients are captured by the SMILeApp and transferred to the hospital where the CC can overview them
	x	x	The CC can access the patient data if agreed to by the patient and share it with the attending physician if needed
eHed		x	The patients and the CC are trained to work respectively with the SMILeApp and SMILeCare applications

*Note: eCCM*, eHealth enhanced Chronic Care Model; *CC*, care coordinator; *SM-S*, self-management support; *DSD*, delivery system design; *CDS,* clinical decision support; *CIS*, clinical information system; *eHed*, eHealth education

### The human-delivered components

The SMILe-ICM’s human-delivered components are 12 face-to-face sessions with an advanced practice nurse (APN)/care coordinator (CC, delivery system design; self-management support). Embedded in the alloSCT team, in addition to coordinating patient care and delivering information and training, the CC provides the desired human factor. Additionally, working closely with the inpatient and outpatient teams, the CC can strengthen the links between the two.

Patients are monitored closely during their inpatient stay, but need to build post-discharge health self-management skills. Immediately post-discharge, they return 4–8 times per month as necessary for follow-up. As their conditions stabilize, follow-up intervals gradually increase to once yearly. As the patients’ first point of contact, the CC delivers all required self-management interventions within (self-management support, delivery system design). All face-to-face sessions will follow a detailed intervention protocol, which guides the intervention delivery and support fidelity among the CCs while delivering the intervention modules. In addition to face-to-face contacts, the CC will also be connected with the patients via the SMILe technology enabling rapid responses to early signs of health deterioration.

### The technology components

The SMILe technology includes the SMILeApp for the patient and the browser-based SMILeCare monitoring component for the care team (delivery system design; clinical information systems). Via the SMILeApp, patients are encouraged to daily enter data for 20 monitoring parameters. These include ratings of general well-being, temperature, weight, and blood pressure measurements, 13 symptom-related parameters (pain; signs of bleeding; nausea; emesis; diarrhea; skin rash; mouth or throat sores; shortness of breath; cough; pain or burning at urination; fatigue, tiredness, or lack of energy; difficulty swallowing; decreased appetite), and three behavioral measures (adherence to infection prevention measures and medication intake, number of steps). According to the data patients enter, they receive automated feedback from the SMILeApp concerning self-management or necessary actions (self-management support; decision support). If one or more parameters exceed pre-defined cut-offs, they receive instructions to contact the transplant center within the next 2 days, as soon as convenient, immediately, or even to call an ambulance immediately.

Furthermore, patients have access to self-management and behavioral support information via a lexicon of complications and parameters assessed by the SMILeApp (self-management support). With the patient’s consent, the data entered are transferred to the university hospital data center, where the CC reviews incoming values and visualizations of their development via the SMILeCare monitoring software (clinical information system). The CC reacts following the SMILe risk-adjusted care protocol (decision support, clinical information systems, delivery system design). Based on the same algorithms that guide the CC, other alloSCT team members will be involved as appropriate. Both patients and CCs receive special SMILe technology training (eHealth education).

During the development phase, the SMILeApp was subjected to two rounds of classical user tests involving 5–6 patients per round, resulting in high user experience ratings [36]. To help the development team clarify design questions (e.g., the logo), and to discuss certain functionalities’ acceptability (e.g., frequency data entry), we also retrieved patient and CC feedback.

- Step: 4 SMILe intervention characteristics and application in daily clinical practice

The three above-described steps resulted in the operational SMILe-ICM’s intervention characteristics ready for roll-out in clinical practice (Fig. 2). The following section describes the intervention’s application in daily clinical practice.

**Fig. 3:**
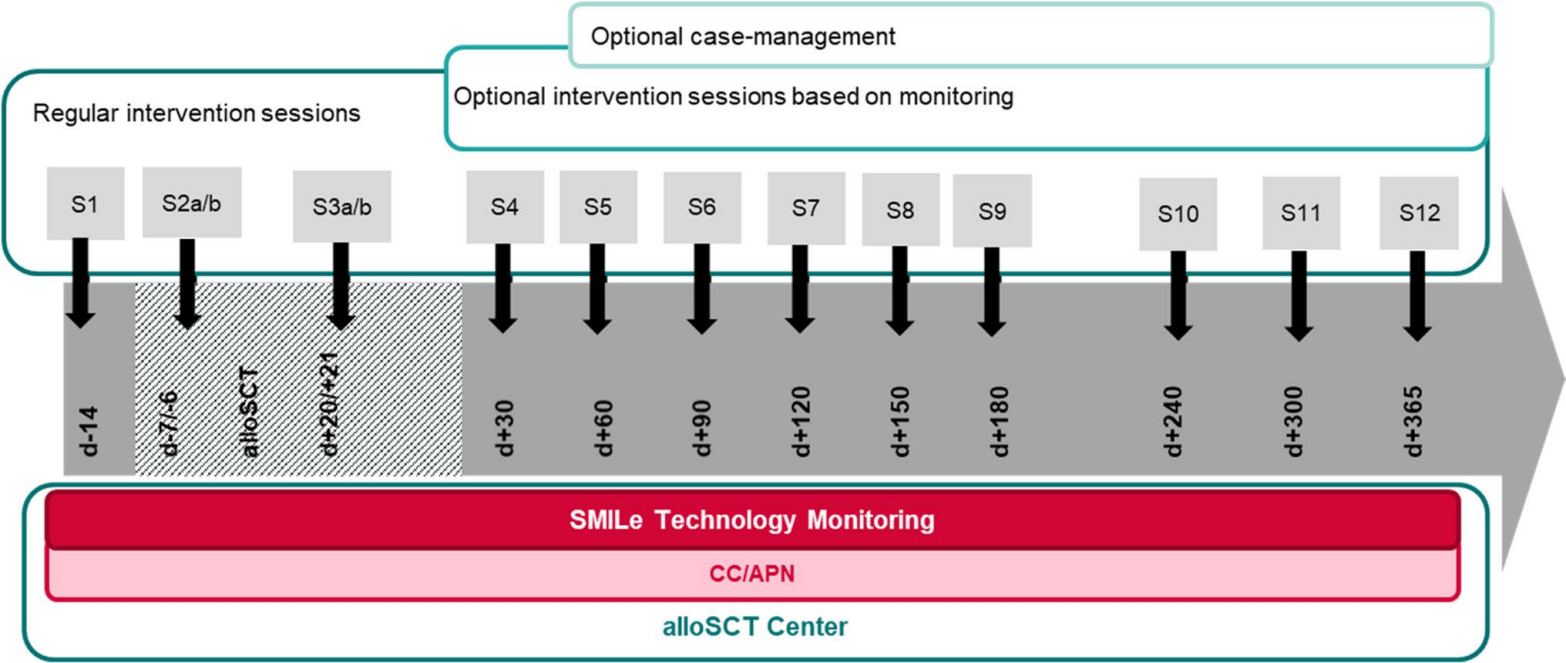
Intervention timing and dosage within the SMILe integrated care model

To recruit patients, the CC contacts patients as soon as they are listed for transplantation (1 to 5 weeks pre-admission), to schedule their first face-to-face session. Patients support needs are highest in the first 6 months post-discharge. Therefore, the CC delivers most interventions between days − 10 pre- and + 180 post-alloSCT in close collaboration with the alloSCT team. Patient contact begins before the inpatient stay and continues via scheduled outpatient clinic appointments as part of usual follow-up care. The CC delivers the highest-frequency intervention dosage over the first 2 months. This usually drops in months 3–6, with the lowest dosage occurring in month’s 7–12 post-alloSCT (Fig. 3). The CC uses the face-to-face sessions to deliver/reiterate oral instructions and/or tailored self-management and behavioral support. Already in the first session pre-alloSCT, patients receive a step counter and the SMILeApp, either installed on their smartphone or on a hospital-provided tablet computer. After teaching them to use the step counter, the CC trains them to enter symptoms (e.g., skin rash) to the app, how to interpret the app’s feedback, and how to react if no feedback is received. While hospitalized, they receive as many SMILeApp training sessions as necessary to use it confidently. Before discharge, to develop the habit of entering their daily data, patients also practice this until proficient.

Depending on the patient’s condition, the intervention protocol allows stepping up of intervention dosage, i.e., for those reporting parameters above cut-off levels or requiring additional support, the CC will immediately contact the responsible clinical team (e.g., treating physician) in the inpatient or outpatient setting based on predefined protocols. Where patients have two or more uncontrolled symptoms, the CC also provides case management (Fig. 3).

## Discussion

This is the first paper to report on the theory-driven, evidence-based development of an eHealth-facilitated alloSCT ICM combining implementation, behavioral, and computer science methods. This includes, respectively, a contextual analysis, the Behavior Change Wheel, and user-centered design informing the agile software development. It provides not only information on this complex intervention’s content, but also step-by-step guidance on how to develop a similar care model for any context and real-world implementation.

Traditional models of care in alloSCT settings are predominantly acute care-driven, with limited focus on self-management**.** Based on prevailing evidence and insights from our contextual analysis, a re-engineering of alloSCT care towards an integrated chronic illness approach is urgently needed. In terms of care coordination and self-management requirements, the first months post-alloSCT are the most complex. These involve high re-hospitalization risks due to various complications—the most frequent being infections and GvHD [2, 6]. EHealth-facilitated ICMs offer promising methods of improving outcomes across a variety of chronic illnesses. However, few have been applied in alloSCT settings, and none has focused on inpatients’ transitions to home—a moment when continuity of care is challenged and complications frequently arise [42]. Such findings are consistent with those of our contextual analysis, which indicated that patients’ greatest support needs occur during the first year post-alloSCT [34].

Numerous commercial eHealth applications are available for cancer settings, some of which are connected to the patients care teams. However, while a small number are either embedded within an ICM approach or are theory-based [18, 28], to our knowledge, these applications have not been developed based on an implementation science approach to ease the adaption, implementation, and sustainability in real-world settings. Consequently, these interventions frequently function as black boxes regarding development, symptoms monitored, or mechanisms underpinning their target behavioral changes. For example, a critical review of 23 eHealth apps for patients with cancer found that no theoretical basis was generally present: only six of the 23 even referred theories or behavior change models [28]. Of the other available offerings, fewer than 20% refer to empirical studies or background source information; only 11.3% are evidence-based; and just under 10.3% involve clinicians in their development processes [43–45]. And while many report user-centered design approaches, few support such claims with insights into end-user involvement or context-specific adaptions. This might result from a strong focus on technology development, with less attention to theoretical underpinnings: such an approach may appear to shorten the path to implementation. In addition, while many applications pay some attention to context-specific requirements, the broader perspective—re-engineering entire care teams and processes—is missing entirely. Overall, alongside the general lack of theoretical underpinnings or context-specific adaptions, the non-use or non-transparent use of user-centered design methodology precludes the uptake and sustained clinical use of virtually any off-the-rack eHealth application in real-world settings.

Furthermore, both Zhao et al. (2016) and Hamel et al. (2019) argue that improving the effectiveness of eHealth design demands the ongoing involvement of all relevant stakeholders to produce a thorough understanding of end-user needs and preferences, with the most effective applications supplying real-time feedback, individualizable elements, and evidence-based medical information [28, 45]. This reflects the findings of our contextual analysis, which revealed that our target patients and clinicians are not interested in a stand-alone eHealth solution, but in a purpose-designed combination of human- and technology-delivered components best reflected by an eHealth-facilitated ICM [34]. It also buttresses the argument that embedding eHealth components in integrated care models facilitates much-needed continuity of care and self-management support between outpatient clinic visits [42].

Our development process included certain notable challenges. Most importantly, implementing an eHealth-facilitated ICM is more than adding an intervention. It is about re-engineering care processes. Within transplant centers, a systematic implementation requires personnel, time-related, and financial resources. Total resource use and associated costs cover all phases, from initial development/adaption to roll out in clinical practice. Indeed, the initial development of the first SMILe-ICM version—starting with a contextual analysis, followed by the content, and software development (within an interdisciplinary team conversant with three methodologies)—took us 2 years [36, 37].

This was time well invested. It allowed us to lay the foundation for a product that fits both the intended context and the target users’ requirements and we believe that this maximizes the likelihood of success when rolling-out and sustaining the intervention in real clinical practice. The preparation of the roll out in clinical practice required team meetings, development/adaption of educational materials, and to secure server space. To operate the SMILe-ICM in clinical practice, we factored in a 100–150% full-time care coordinator position, plus yearly server and software maintenance costs. Specific cost details are context specific. Our currently running hybrid-1 effectiveness-implementation RCT of the first SMILe–ICM will be followed by a full economic analysis. This will include the patients’ medical resource use over a 1-year follow-up and will be weighed against those costs of operating the SMILe-ICM intervention.

The SMILe-ICM trial’s full results will provide further clinical and implementation outcomes for a first center in Germany. We have already adapted it to a Swiss center, where we are currently preparing for the adapted version’s implementation and testing phase. Even after the final evaluation, this will allow continuing insights into adaption costs.

Limitations of our human and time resources prevented the completion of the digitalization process as originally planned. A serious regulatory barrier was the main reason for this: The European Medical Device Regulation (MDR; EU2017/745) defines the originally planned SMILeApp as a class 2b medical device, necessitating, e.g., tightly regulated development, certification, and ongoing quality management. Unable to meet these requirements, our academic project team was forced to cut back the planned level of digitization. While the monitoring and follow-up module is now digitalized with intermitted automated feedback and decision support, patients receive these information’s as written leaflets. The other modules are still purely in a face-to-face delivering mode. In parallel of testing the SMILe-ICM, following all regulatory requirements (MDR; EU2017/745), the digitalization process of the eHealth components facilitating the face-to-face components will continue until all module components are digitalized and compliant with class 2b medical device regulations. However, testing the system at an early stage provides insights influencing the creation of future modules; and we believe that the methods used increase the probability of sustainable implementation and acceptance in real-world clinical practice, while reducing research resource waste.

## Conclusions

We found that the alloSCT setting would benefit strongly from the re-engineering of its care teams and processes towards an eHealth-facilitated ICM. Despite high levels of activity in the eHealth sector, though, empirical evidence is scarce. Many available eHealth applications are neither embedded within an ICM nor offer information regarding their theoretical underpinnings nor explanations of how contextual factors, end-user involvement, or effective behavior change techniques were integrated into their development processes. With this report, in addition to providing step-by-step guidance for development of an eHealth-facilitated ICM, we describe how the resulting model can be integrated within alloSCT patient care. We developed the SMILe-ICM via an iterative process merging implementation, behavioral, and computer science methods. This combination facilitates the development and tailoring of meaningful theory- and evidence-based interventions to end users’ context-specific needs and preferences, thereby giving the resulting interventions the highest possible chance of uptake and sustained use.

## Supplementary Information

Below is the link to the electronic supplementary material.

Supplementary file1 (DOCX 60.0 KB)

## Data Availability

N/A Code availability The SMILe technology is still in a testing and developing face; software codes cannot be made available yet.
