# Supplementary material for: Development of an integrated model of care for allogeneic stem cell transplantation facilitated by eHealth—the SMILe study
Source: Support Care Cancer. 2021 Jul 5;29(12):8045–57. doi: 10.1007/s00520-021-06328-0 (PMC8550349; doi:10.1007/s00520-021-06328-0)
Supplement: Supplementary file 1 — Supplementary file1 (DOCX 60.0 KB) [file 520_2021_6328_MOESM1_ESM.docx]

Supplementary file for:

# Development of an Integrated Model of Care for allogeneic SteM cell transplantatIon faciLitated by eHealth – The SMILe Study

Supportive Care in Cancer

Lynn Leppla ^1, 2^; Anja Schmid ^1,2^; Sabine Valenta^1,3^, Juliane Mielke ^1^; Sonja Beckmann ^1,4^; Janette Ribaut ^1,4^; Alexandra Teynor ^5^, Fabienne Dobbels ^6^; Nathalie Duerinckx ^6^; Robert Zeiser ^2^, Monika Engelhardt ^2^; Sabine Gerull ^3^, Sabina De Geest ^1, 6^ *on behalf of the* *SMILe study team*

^1^ Institute of Nursing Science, Department Public Health, University of Basel, Switzerland

^2^ Department of Medicine I, Medical Center – University of Freiburg, Faculty of Medicine, University of Freiburg, Germany

^3^ Department of Hematology, University Hospital Basel, Switzerland

^4^ Center of Clinical Nursing Science, University Hospital Zurich, Switzerland

^5^ Faculty of Computer Science, University of Applied Sciences Augsburg, Germany

^6^ Academic Centre for Nursing and Midwifery, Department of Public Health and Primary Care, KU Leuven, Belgium

**Corresponding author**: Sabina De Geest, PhD, RN, Nursing Science (INS), Department Public Health (DPH), Faculty of Medicine, University of Basel, Bernoullistrasse 28, CH-4056 Basel, Switzerland, Phone: +41 61 2070951, Fax. +41 61 207 09 55. Email: sabina.degeest@unibas.ch

**Abbreviations:**

BCT=Behavior Change Technique;

COM-B= Capability, Opportunity Motivation Behavior;

TDF=Theoretical Domains Framework;

Domains: Knowledge=K; Skills=S; Memory attention, decision processes=MAD; Behavioral regulation=Br; Social influences=Si; Environmental context and resources= Env; Emotion=Em; Intension= Int; Beliefs about consequences= B cons; Beliefs about capabilities= B cap; Optimism= O; Goals= G; Social/professional role and identity= Id; Reinforcement=Reinf..

| **Supplementary Table 1:** Description of the SMILe intervention content and mechanism of change using the Behavior-Change-Wheel [1] | | | | | | | |
| --- | --- | --- | --- | --- | --- | --- | --- |
| ***Module: Monitoring & Follow-up*** | | | | | | | |
| **Target behavior** | **Problem based on context analysis and evidence** | **Content to tackle the problem** | | | **Mechanism of change** | | |
|  |  | **Mode of delivery**  Human &Technology components | **User-Story**  (As a…I want…so that) | **BCTs** | **Functions** | **COM-B** | **TDF** |
| **Improved recognition, evaluation and acting upon symptom** | - Uncertainty about symptom assessment [2]. | - CC explains & trains symptom assessment & SMILeApp use. - CC evaluates SMILeApp use with patients and care-givers during visits. - SMILeApp supports symptom assessment. - SMILeCare facilitates symptom monitoring by CC and praises patients in visits. | As a patient I want a system to assess symptoms so that I feel more secure.  As a CC I want a system to monitor symptoms of patients so that I can detect complications early. | 9.1 Credible source  4.1 Instruction perform the behavior  5.1 Info health consequences  6.1 Demonstration of the behavior  2.2 Feedback on behavior  2.7 Feedback outcome of behavior  8.1 Behavioral practice  15.1 Verbal persuasion  2.3 Self-Monitoring of behavior  12.5 Adding objects  2.5 Monitor outcome behavior by others without feedback  10.4 Social reward | Training  Persuasion | **Physical Capability** | S |
|  | - Uncertainty about symptom judgement and reporting [2]. - High cognitive dysfunction and fatigue levels in alloSCT patients [3]. - Impaired retrospective recalling of symptoms [4]. - Improved survival, quality of life and lower re-admission rate through electronic symptom monitoring with self-management support [5,6]. | - CC explains critical symptoms & trains how to react if problems occur with patients and care-givers. - CC discusses frequency of SMILeApp use by patients and if they managed to enter data as agreed upon. - CC provides feedback about development of parameters and outcomes. - SMILeApp provides feedback on severity of symptoms & how to act upon. - SMILeApp provides a lexicon with self-management instructions. | As a patient I want a daily reminder for using the system so that I do not forget to enter my data.  As a patient, I want a feedback on my self-assessed vital signs and symptoms so that I have support in my self-management and decision making.  As a patient, I want written information 24/7 available so that I can look up discussed information. | 9.1 Credible source  4.1 Instruction perform the behavior  5.1 Info health consequences  6.1 Demonstration of the behavior  8.1 Behavioral practice  7.1 Prompts/ cues  1.2 Problem solving  3.1 Social support  1.1 Goal setting  8.3 Habit formation  1.4 Action planning  1.5 Review goals (behavioral)  2.6 Biofeedback  2.7 Feedback outcome of behavior  5.1 Info health consequences | Education  Training  Enablement | **Psychological Capability** | K  MAD  Br |
| **Improved recognition, evaluation and acting upon symptoms** | - Patients and clinicians would benefit from monitoring of critical symptoms, - No system available, - >70% of patients would share their da or use a App from hospital [2]. - Remote monitoring of symptoms improves survival [6]. | - SMILeApp provides opportunity to monitor symptoms. - CC encourages daily use of SMILeApp, identifies barriers to use it and set goals with patients and care-givers. | As a patient I want to have the option to assess and share my entered data so that I have the certainty that someone is watching over me. | 12.5 Adding objects  1.2 Problem solving  1.1 Goal setting  1.4 Action planning | Training  Enablement | **Physical Opportunity** | Env |
|  | - The knowledge that someone watches over your parameters gives a feeling of security, - Value of social support by peers and family, - Patients rate the importance of having technologies to share their data with others with a median of 8 (0-10)[2]. | - CC offers 12 face-to-face sessions over the first year post-alloSCT and cares for patients and families. - SMILeCare connects patients virtually to CC and allows to overview incoming values. - Patients observe other patients, family members using Apps for their health. | As a CC, I want a system to monitor important parameters of patients at home within the hospital so that I can detect complications early. | 6.1 Demonstration of the behavior  12.5 Adding objects | Modelling  Enablement | **Social opportunity** | Si |
|  | - Clinicians assume that patients might be more anxious when assessing symptoms at a regular basis, - Might increase contacts to hospital, - Patients feel secured and watched over, - Would value a system tracking their parameters [2]. | - CC reviews together with patients the use of the SMILeApp in each face-to-face session. - CC praises the use of the SMILeApp. - CC teach patients to leave their smartphone next to their bed as a reminder to enter parameters. - SMILeApp provides an overview about patents parameters development over time so that they can observe changes. | As a CC, I want an overview how frequent patients entered their data into the system so that I can give feedback.  As a patient I want positive feedback when I use the system on a regular basis so that I keep motivated.  As a patient I want an overview about my entered data so that I can see changes over time and feel motivated to continue. | 2.2 Feedback on behavior  10.4 Social reward  7.1 Prompt and cues  8.3 Habit formation | Incentivisation  Training  Enablement | **Automatic Motivation** | Em |
|  | - Patients are affright to get re-hospitalized, - Patients believe that monitoring of medical parameters is important [2,7]. - Early recognition decreases re-hospitalization, costs and prevents co-morbidities [8]. | - CC discusses with patients and care-givers that monitoring of symptoms can help to detect complications early and may improve long-term outcomes - CC offers patients and care-givers to call in terms of insecurity | As a patient, I want to have contact information within the system so that I know who to contact. | 5.3 Info social/environmental cons  5.1 Info health consequences | Education | **Reflective Motivation** | B Cap  O  Id |

*Note:* BCT=Behavior Change Technique; COM-B= Capability, Opportunity Motivation Behavior; TDF=Theoretical Domains Framework; domains: Knowledge=K; Skills=S; Memory attention, decision processes=MAD; Behavioral regulation=Br; Social influences=Si; Environmental context and resources= Env; Emotion=Em; Intension= Int; Beliefs about consequences= B cons; Beliefs about capabilities= B cap; Optimism= O; Goals= G; Social/professional role and identity= Id; Reinforcement=Reinf..

| ***Module: Infection prevention*** | | | | | | | |
| --- | --- | --- | --- | --- | --- | --- | --- |
| **Target behavior** | **Problem based on context analysis and evidence** | **Content to tackle the problem** | | | **Mechanism of change** | | |
|  |  | **Mode of delivery**  Human &Technology components | **User-Story**  (As a…I want…so that) | **BCTs** | **Functions** | **COM-B** | **TDF** |
| **Adequate Hand Hygiene** | - Patients need to apply good hand hygiene at a daily basis to prevent infections as they are immune compromised [9] - Patients feel overwhelmed by recommendations about hand hygiene [2] | - CC trains patients and care-givers how to perform the correct sequence of hand disinfection and when to apply handwashing and disinfection and how to care for their skin. | As a patient I want to have information about hand disinfection so that I can refresh my knowledge at any time. | 4.1 Instructions perform behavior | Enablement  Training | **Physical Capability** | S |
|  | - Lack of knowledge and awareness in terms of risk of infections via contamination. - Reduced awareness of impact of hand-disinfection to reduce infections [10,11] - Memory problems in terms of opportunities [2] when to apply adequate hand hygiene. | - CC provides oral, written and visual instructions about risks of infections, procedural knowledge and opportunities of hand disinfection: correct fluids, dosing of fluids, duration and coverage of hand surfaces and demonstrates the correct performance. - CC trains hand-disinfection and gives performance feedback using UV light box with fluorescent test lotion. - CC discusses barriers and supports to overcome them. - Goals will be set at each visit in terms of hand-disinfection and reviewed the next time - CC asks at each follow-up session about behavior performance and monitors for infection. - SMILeApp provides information about hand-disinfection. |  | 4.1 Instructions perform behavior  5.1 Info health consequences  11.3 Conserving mental resources  2.2 Feedback on behavior  1.2 Problem solving  6.1 Demonstration of the behavior  8.7 Graded tasks  8.6 Generalization target behavior  1.1 Goal setting  1.4 Action planning  2.6 Biofeedback  1.5 Review behavior goals  8.1 Behavioral practice/rehearsal | Education  Modelling  Training  Enablement  Environmental restructuring | **Psychological Capability** | K  MAD  Br |
|  | - Lack of products and inadequate supplies [10] for adequate hand hygiene. | - CC provides mini dispensers of hand-disinfection fluids which they can always carry with them. - SMILeApp sends reminders for important hand-disinfection moments. | As a patient I want to be reminded to perform hand disinfection at important moments so that I do not miss it. | 12.5 Adding objects  7.1 Prompts, cues | Environmental restructuring | **Physical Opportunity** | Env |

| **Adequate Hand Hygiene** | - Lack of culture of personal accountability for performing hand-disinfection [10]. - Lack of awareness of own responsibility [2] of adequate hand hygiene. | - CC provides oral and written information about the association of hand-disinfection and infections. - That it is a common and supportive behavior when being immunocompromised. - CC encourages care-givers to support patients. - CC refers to SMILeApp where the hand-disinfection sequence can be looked up. | As a CC I want to have information about hand disinfection so that I can refer to that. | 5.3 Info social/environmental cons  5.1 Info health consequences  6.3 Info others approval  6.1 Demonstration of the behavior  3.1 Social support (unspecific) | Modelling  Persuasion  Education  Enablement | **Social opportunity** | Si |
| --- | --- | --- | --- | --- | --- | --- | --- |
|  | - High cognitive dysfunction and fatigue levels in alloSCT patients [3]. - Smell of fluids is associated with hospital and illness and might result in avoidance. | - CC offers different fluids, information about hand-disinfection and praises patients when they reached their goals. - Encourages developing habits. - SMILeApp sends pop-up questions once weekly to ask for hand-disinfection adherence and praises if successful. - SMILeCare displays answers in terms of adherence. | As a patient, I want to record how well I managed to disinfect my hands so that I can monitor my performance.  As a patient I want to receive a feedback when I have been adherent so that I feel motivated to continue.  As a CC I want to have an alarm if the patients entered two non-adherent answers so that I can discuss this with the patients. | 4.1 Instructions perform behavior  2.3 Self-monitoring of behavior  10.4 Social reward  2.5 Self-monitoring outcomes of behavior by others without feedback  8.3 Habit formation  12.5 Adding objects | Persuasion | **Automatic Motivation** | E |
|  | - Belief that there is insufficient evidence that hand-disinfection adherence reduces infection [10]. - Belief that it is needed in hospital but not at home [2]. | - CC supports the gain-framing toward a shared responsibility in caring for their family’s well-being (rather than a cleansing act). - Patients and care-givers reflect and compare reasons for wanting and not wanting to disinfect their hands. - CC prompts with patient a concrete planning of a self-reward for staying committed until the next session. - CC praises patient and caregiver for their performance and assertion that they can and will succeed in preventing infections effectively via hand disinfection. |  | 13.2 Framing/Reframing  9.2 Pros an cons  10.9 Self-reward  15.1 Verbal persuasion capability | Enablement  Persuasion | **Reflective Motivation** | B Con  B Ca  Int |

*Note:* BCT=Behavior Change Technique; COM-B= Capability, Opportunity Motivation Behavior; TDF=Theoretical Domains Framework; domains: Knowledge=K; Skills=S; Memory attention, decision processes=MAD; Behavioral regulation=Br; Social influences=Si; Environmental context and resources= Env; Emotion=Em; Intension= Int; Beliefs about consequences= B cons; Beliefs about capabilities= B cap; Optimism= O; Goals= G; Social/professional role and identity= Id; Reinforcement=Reinf..

| ***Module: Infection prevention*** | | | | | | | |
| --- | --- | --- | --- | --- | --- | --- | --- |
| **Target behavior** | **Problem based on context analysis and evidence** | **Content to tackle the problem** | | | **Mechanism of change** | | |
|  |  | **Mode of delivery**  Human &Technology components | **User-Story**  (As a…I want…so that) | **BCTs** | **Functions** | **COM-B** | **TDF** |
| **Reducing exposure risk to airborne pathogens** | - Lack of skills how to wear a FFP2 mask correctly. | - CC instructs patients and caregivers when and how to wear FFP2 masks. - Demonstrates the correct handling and let patients practice. | As a CC I want to have information about airborne pathogens and mask wearing so that I can refer to this if I think patients lack the knowledge. | 4.1 Instructions perform behavior  6.1 Demonstration of the behavior  8.1 Behavioral practice/rehearsal | Enablement  Training | **Physical Capability** | S |
|  | - Lack of knowledge about respiratory infections and how to reduce exposure risk [12]. - Limited ability to retain information due to cognitive impairments and fatigue [2]. | - CC provides oral, written and visual instructions about exposure risks of airborne pathogens and correct mask wearing. - SMILeApp asks once weekly if patients managed to apply the exposure risk reduction for airborne pathogens e.g. mask wearing, avoiding crowds of people. - SMILeCare displays answers in terms of adherence so that CC can give feedback at each visit. | As a patient, I want to record how well I managed to wear my mask so that I can monitor my performance.  As a patient I want to receive a feedback when I have been adherent so that I feel motivated to continue.  As a CC I want to have an alarm if the patients entered two non-adherent answers so that I can discuss this with the patients. | 5.1 Info health consequences  2.2 Feedback on behavior  11.3 Conserving mental resources  2.3 Self-monitoring of behavior | Education  Enablement | **Psychological Capability** | K  MAD |
|  | - FFP2 masks are not always available in the outpatient setting. - Lack of knowledge in terms of exposed situations and how to avoid them. | - CC provides a set of 3 masks and advice where to buy new ones. - CC explains how to change physical environment in order to reduce risk exposure. - Informs that infection prevention as a socio-cultural norm is not that established as it is in other countries. |  | 12.1 Restructuring environment  5.3 Info social/environmental cons  12.5 Adding objects | Environmental  Restructuring  Enablement  Training | **Physical Opportunity** | Env |
|  | - Wearing masks in exposed situations is not common in European countries [13]. - Lack of social acceptance and role models [13]. - Low clinician support due to reduced belief in effectiveness of masks [13]. | - CC provides social support to follow recommendations and advises to exchanges with other patients. - CC informs caregivers how to support the patient following recommendations reducing exposure risk. - CC discusses pros and cons of recommendations. |  | 3.1 Social support (unspecified)  6.2 Social comparison  9.2 Pros and cons | Modelling  Environmental restructuring  Enablement | **Social Opportunity** | Si |
| **Reducing exposure risk to airborne pathogens** | - Fear of stigmatization. - Negative emotions as fear, anxiety or feelings of constriction when wearing the mask. - No established habits to avoid exposes situations. | - CC encourages to reward self in future with valued objects if patient has adhered to recommendations reducing exposure risk. - CC praises patient if he stays free of respiratory infections and/or managed to adhere to recommended behavior. - CC assesses patient`s emotions after performing exposure risk reduction (e.g. wearing a mask). |  | 10.9 Self-reward  10.4 Social reward  11.2 Reduce negative emotions  5.4 Monitoring emot. consequences | Environmental restructuring  Modelling  Enablement | **Automatic Motivation** | Em |
|  | - Patients have false beliefs about consequences and capabilities [2]. | - CC reviews the set goals at each follow-up visit. - CC discusses discrepancies of goals and current behavior. - Patients identifies barriers and discuss strategies how to overcome barriers. - CC prompts the mask wearing for the most critical situations as e.g. sitting in outpatient waiting rooms, using public traffic. - Patients affirm when and where to wear a mask or to follow other recommendations reducing exposure risk. - CC points out that he/she beliefs in patients’ ability to successfully reduce exposure risk to airborne pathogens in daily life. |  | 1.3 Goal setting  1.5 review behavior goals  1.6 Discrepancy behavior and goal  1.2 Problem solving  1.4 Action planning  1.9 Commitment  15.1 Verbal persuasion capability  9.1 Credible source | Education  Persuasion | **Reflective Motivation** | Int |

*Note:* BCT=Behavior Change Technique; COM-B= Capability, Opportunity Motivation Behavior; TDF=Theoretical Domains Framework; domains: Knowledge=K; Skills=S; Memory attention, decision processes=MAD; Behavioral regulation=Br; Social influences=Si; Environmental context and resources= Env; Emotion=Em; Intension= Int; Beliefs about consequences= B cons; Beliefs about capabilities= B cap; Optimism= O; Goals= G; Social/professional role and identity= Id; Reinforcement=Reinf..

| ***Module: Infection prevention*** | | | | | | | |
| --- | --- | --- | --- | --- | --- | --- | --- |
| **Target behavior** | **Problem based on context analysis and evidence** | **Content to tackle the problem** | | | **Mechanism of change** | | |
|  |  | **Mode of delivery**  Human &Technology components | **User-Story**  (As a…I want…so that) | **BCTs** | **Functions** | **COM-B** | **TDF** |
| **Safe handling, preparation and consumption of food** | - Long-term impaired cognitive function, fatigue or nausea and emesis hampering the correct selection, preparation and handing of food at home [3]. | - CC provides oral, written and visual information about which food to avoid, and what to pay attention to when buying/selecting food. - CC informs that these rules change depending on the immune status. - CC checks knowledge on which food is safe to eat and shows patients and caregivers where to look up information in the SMILeApp. - At each follow-up visit goals will be reviewed. | As a patient I want to have information about which food to avoid and how to adapt rules so that I feel secure when buying food. | 4.1 Instructions perform behavior  12.5 Adding objects  1.2 Problem solving  1.5 Review behavior goals  11.3 Conserving mental resources | Enablement | **Physical Capability** | S |
|  | - Problems in understanding why safe food selection and handling is important, and apply it to his/her own situation [14]. - Patient have low cognitive capacity to be able to process and remember all the instructions [2]. | - CC provides information on risk and consequences of foodborne/waterborne & fecal-oral pathogen infections. - CC mentions that she is very pleased to see that no infections occurred. - Patients get encouraged to watch the videos at the SMILeApp about how to clean used kitchen utensils after usage. - CC discusses an agreed goal - CC askes patient to repeat the recommended food and explain how he/she would buy, store, handle food and clean kitchen utensils. | As a patient I want to have instructions how to clean kitchen utensils after usage so that I`m more confident in preparing and handling food. | 5.1 Info health consequences  2.7 Feedback outcomes behavior  6.3 Info others approval  10.4 Social reward  6.1 Demonstration of the behavior  1.1 Goal setting  8.1 Behavioral practice/rehearsal | Education  Enablement | **Psychological Capability** | K  MAD |
|  | - Lack of awareness how and where to buy safe food and where to get household help [15]. - Frequently inadequate equipment to safely store and prepare food [15]. | - CC instructs how to buy (e.g. which food/which condition), store (e.g. stored in boxes in a fridge with 6 °C) and handle food when cooking (e.g. which cutting boards). - Discusses strategies if patients are not able e.g. organizing household help. - CC and SMILeApp demonstrates how to handle kitchen utensils after use e.g. cutting boards | As a patient I want to have instructions how to store and handle food so that I`m more confident in preparing and handling food. | 12.5 Adding objects  12.1 Restructuring environment  3.1 Social support (unspecific)  1.2 Problem solving  6.1 Demonstration of the behavior  4.1 Instructions perform behavior | Environmental  Restructuring  Enablement  Training | **Physical Opportunity** | Env |
| **Safe handling, preparation and consumption of food** | - The patient’s environment understands why safe food selection and handling is important. - Patients’ needs people they can rely on to do the shopping when not feeling well or when not being capable to go shopping themselves - If others prepare meals, they need to follow the same rules regarding safe food handling, storage and preparation. | - CC provides oral, written and visual information for care-givers or other supporting people about which food to avoid, and what to pay attention to when buying/selecting food. - A bullet point sheet or visual aid will be given to the patient, to keep in his/her purse; the purse of support people, or to hang on the fridge or in the kitchen as visual aids. |  | 5.1 Info health consequences  7.1 Prompts, cues  3.2 Social support (practical) | Modelling  Environmental restructuring  Enablement  Education | **Social Opportunity** | Si |
|  | - Patients need to form a habit in always carefully reflecting on whether certain food is safe to buy and to eat and form a habit in safely storing, handling and preparing food. - Patients might feel overwhelmed or depressed when learning about all these instructions - Eating safe food does not evoke negative emotions (e.g. it reminds of not being healthy; missing favorite foods (e.g. raw meat). | - CC explains that she/he will monitor how well the patient succeeds in following rules, by asking 4 questions on a weekly basis via the SMILeApp so that feedback can be given. - CC asks patients to memorize the list, arranges for another meeting and informs that patient can ask questions in case some info is not clear. - CC explores how the patient can reward himself/herself regularly at home for following the rules. | As a CC I want to see how well patients managed following the rules so that I can act upon that. | 2.2 Feedback on behavior  8.1 Behavioral practice/rehearsal  8.3 Habit formation  10.9 Self-reward | Enablement | **Automatic Motivation** | Em |
|  | - Patients need to understand/ believe that these strict rules are important for his/her health so that infections can be avoided. - Patients need to believe in one’s capability Patient needs to be optimistic that desired goal (i.e. avoidance of infections) will be attained if the rules are followed strictly. - Patients need to express the intention to always trying to apply the rules on safe food selection, handling and preparation. | - CC stresses that it is normal that this is a lot of information, but that patient will be capable of following these and that CC is there to answer questions. - CC acknowledges that this might sound overwhelming, but she/he trusts the patient will be successful and beliefs the patient has the capacity to do a great job. - CC identifies together with patient barriers for not following the rules and discuss strategies how to overcome barriers. |  | 3.3 Social Support (emotional)  9.1 Credible source  15.1 Verbal persuasion capability  1.2 Problem solving | Education | **Reflective Motivation** | B cons  Id |

*Note:* BCT=Behavior Change Technique; COM-B= Capability, Opportunity Motivation Behavior; TDF=Theoretical Domains Framework; domains: Knowledge=K; Skills=S; Memory attention, decision processes=MAD; Behavioral regulation=Br; Social influences=Si; Environmental context and resources= Env; Emotion=Em; Intension= Int; Beliefs about consequences= B cons; Beliefs about capabilities= B cap; Optimism= O; Goals= G; Social/professional role and identity= Id; Reinforcement=Reinf..

| ***Module: Medication Adherence*** | | | | | | | |
| --- | --- | --- | --- | --- | --- | --- | --- |
| **Target behavior** | **Problem based on context analysis and evidence** | **Content to tackle the problem** | | | **Mechanism of change** | | |
|  |  | **Mode of delivery**  Human &Technology components | **User-Story**  (As a…I want…so that) | **BCTs** | **Functions** | **COM-B** | **TDF** |
| **Correct taking and timing of immunosuppressive medication** | - High symptom burden of nausea and emesis, decreased cognitive functioning after alloSCT [3,16]. - Poor physical condition post transplantation [17,2]. | - Patients will be encouraged to prepare a plan how to deal with barriers and practice how to react at home (e.g. taking antiemetics before other medication). - CC demonstrates how to prepare medication and when to take e.g. antiemetic’s. | As a patient I want to have reliable information how to plan my expected actions (e.g. taking medication while having nausea) so that I do not forget the necessary preparations. | 1.4 Action planning  6.1 Demonstration of the behavior | Enablement | **Physical Capability** | S |
|  | - Lack of knowledge about medication and consequences of non-adherence [18,19]. - Lack of routine and information overload [2,20] - No sense of autonomy regarding medication intake [20] - Forgetfulness and forgetting get a new prescription on time [17,18] - Unable to cope with changed prescription [21]. | - CC provides oral, written and visual information about effects and side-effects of medication as well as consequences of non-/adherence and refers to SMILeApp. - SMILeApp asks patient to document and confirm medication intake twice daily. - CC provides feedback about medication intake. - CC informs about blood level of immunosuppressives, reference range and signs of acute rejection. - CC trains and practices with the patient and caregiver how to read and prepare the medication plan. - CC prompts patients to combine medication intake with another habit (e.g., eating breakfast). - CC asks caregivers to support correct intake of medication by reminding, preparing or filling in the prescriptions. - Goals will be set together with patient to take the medication correctly with a deviation < 2hours in 95% of the time. - Review of goal in terms of reach and discrepancies. - Identification of barriers and discussion of strategies. | As a patient I want to find information on what my medication is for and will happen, if I do (not) take it as prescribed (incl. wrong time) so that I know the importance of doing it correctly.  As a patient I want to get explained how to use the electronic medication plan so I can check when I have forgotten.  As a patient I want to know how to prepare my medication so that I can do it on my own correctly.  As a patient I want to self-monitor whether I take my medication as prescribed so that I know whether I take the drugs correctly.  As a patient I want to get feedback whether I take my medication sufficiently as prescribed so that I can be sure that I take the medication correctly  As a patient I want to be reminded of my set goals (which were set during visit) on a self-determined interval (e.g. daily / once a week) so that I am aware of my goal and know what to target at. | 5.1 Info health consequences  11.3 Conserving mental resources  2.3 Self-monitoring of behavior  2.2 Feedback on behavior  2.7 Feedback outcomes behavior  4.1 Instructions perform behavior  8.1 Behavioral practice/rehearsal  8.3 Habit formation  3.2 Social support (practical)  1.1 Goal setting  1.5 Review behavior goals  1.6 Discrepancy behavior and goal  1.2 Problem solving | Education  Enablement  Training | **Psychological Capability** | K  MAD  Br |
| **Correct taking and timing of immunosuppressive medication** | - Not having medicines when being away from home [21,17]. - Time of intake «does not fit» to lifestyle, - Interruptions in daily routine, - Longer time since transplantation [17,2,22]. | - CC recommends to use practicable weekly pill boxes and the SMILeApp for looking up and documenting medication intake and time. - CC instructs to store medication in clear visible places (e.g., next to the coffee machine), to prompt medication intake and to use SMILeApp for reminders. - CC instructs patients to always have travel packs of medications prepared. - Identification of barriers and discussion of strategies. | As a patient I want to have my medication plan in the system so that I can look up my medication.  As a patient I want to be able to update the medication plan in the system when the prescription of physician changes so that I have a current medication plan.  As a patient I want to get a reminder when I need to take my medication so that I don’t forget to take it. | 12.5 Adding objects  7.1 Prompts, cues  4.1 Instructions perform behavior | Environmental  Restructuring  Enablement  Training | **Physical Opportunity** | Env |
|  | - Lack of family/social support [20,22]. - Avoiding taking medication in public / in front of friends, - Lack of positive and negative role models [21]. - Lack of individual support (by nurses, pharmacists) [18]. | - CC invites caregivers to be part of the intervention sessions and to support patients in the correct intake of medication - CC recommends to look for a self-help group / peer website to talk about strategies for medication intake if problems occur. |  | 9.1 Credible source  3.1 Social support (unspecified)  3.2 social support (practical) | Training | **Social Opportunity** | Si |
|  | - Feeling overwhelmed [21,2]. - Burnout / treatment fatigue [18]. - Negative emotions / attitude [20] . - Desire for independence in self-management [22]. - Incompatibility of the IS, - Side effects [22,2,20]. | - Patients explore difficult circumstances in which they nevertheless managed to take their medication. - CC tells the patient that he/she can successfully perform the behavior. - CC tells the patient that he/she can always contact her in case of difficulties. - SMILeApp allows to document and reminds of medication intake and time. | As a patient I want to be able to record occasions with correct medication intake in the system so that I feel confident to be successful again.  As a patient I want to get a motivational feedback that I  can successfully perform the behavior so that I feel capable to manage the correct medication intake.  As a patient I want to have the opportunity to call a qualified health care provider if there are unexpected barriers (which were not discussed face-to-face) so that I get support in challenging situations.  As a patient I want to customize the system (e.g. different tones, signals, colors, pictures) so that I connect medication intake with a positive feeling  As a patient I want to be able to signal the CC that she/he should call back when she/he has time so that I get help without disturbing the CC in an unsuitable situation. | 15.3 Focus on past success  15.1 Verbal persuasion capability  15.1. Written persuasion capability  7.1 Prompts, cues  3.3 Social Support (emotional)  12.5 Adding objects  1.2 Problem solving | Persuasion  Environmental restructuring  Training  Enablement | **Automatic Motivation** | Em  Reinf |
|  | - Lack of intention to adhere [17]. - Not interested in learning about medication before transplant [21]. - Beliefs in illness, medication and side effects [17] - No confidence in self-management [18] - Lack of problem solving competence & self-efficacy [23] | - Patient list and compare the advantages and disadvantages of taking the medication correctly. - CC point out if the recorded number / time of medication intake does not fit to the set goal. - CC praises patient performance if the medication intake was always correct in time. - SMILeApp provides feedback when set goals are not reached. | As a patient I want to get a signal if the recorded number / time of medication intake does not fit to the goal set so that I realize that I have to change my behavior. | 9.2 Pros and cons  1.6 Discrepancy behavior and goal  10.4 Social reward | Education  Incentivisation | **Reflective Motivation** | Int  B cons  B cap  G |

*Note:* BCT=Behavior Change Technique; COM-B= Capability, Opportunity Motivation Behavior; TDF=Theoretical Domains Framework; domains: Knowledge=K; Skills=S; Memory attention, decision processes=MAD; Behavioral regulation=Br; Social influences=Si; Environmental context and resources= Env; Emotion=Em; Intension= Int; Beliefs about consequences= B cons; Beliefs about capabilities= B cap; Optimism= O; Goals= G; Social/professional role and identity= Id; Reinforcement=Reinf..

| ***Module: Physical Activity*** | | | | | | | |
| --- | --- | --- | --- | --- | --- | --- | --- |
| **Target behavior** | **Problem based on context analysis and evidence** | **Content to tackle the problem** | | | **Mechanism of change** | | |
|  |  | **Mode of delivery**  Human &Technology components | **User-Story**  (As a…I want…so that) | **BCTs** | **Functions** | **COM-B** | **TDF** |
| **Reducing Sedentary Bouts** | - Increased levels of fatigue and physical weakness in the first weeks after alloSCT [2]. - MET < 3 decrease, MET >3 increases after alloSCT [24] - Physical inactivity 27%-85% [25] | - CC provides oral and written information about breaking sedentary bouts and explains the concepts of physical activity. Shows health benefits and recommended activities and suggests intervals for breaking sedentary bouts: 3/30 min or 6/60 min. - CC explains with examples how sedentary bouts can be reduced during in- and outpatient time (e.g., walking at the hospital ward) - CC praises the patient if the goal was reached. - SMILeApp collects number of daily steps and reminds once daily. - SMILeApp shows daily steps and sends graphical feedback on behavior compared to outcome goal. - CC encourages patients to set a higher/lower step goal of +/- 500 steps if they did not manage to reach their goal > 3 days. | As a patient I want to document my number of daily steps so that I can see my performance.  As a patient I want to have a visual feedback on my daily steps in comparison to my defined goal so that I can see if I was successful in reaching my goal.  As a patient I want to have a reminder in the morning so that I can enter my number of steps performed on the previous day. | 5.1 Info health consequences  4.1 Instructions perform behavior  2.2 Feedback on behavior  2.6 Biofeedback  2.3 Self-monitoring of behavior  7.1 Prompts, cues  8.7 Graded tasks | Education  Training | **Psychological Capability** | K  MAD  Br  S |
|  | - Patients have the opportunity to walk. - 50% are open to use electronic devices also for counting steps and to promote health behavior [2]. | - Patient receives a step counter as an indirect measure of breaking sedentary bouts. - CC encourages the patient to enter the daily steps reached into the SMILeApp. - CC explains the step counter functions, activating step count function, check number of daily steps, setting reminder alarm. Advise to wear the tracker in a position where it also counts steps while cycling and show how to enter the steps into the app. |  | 12.5 Adding objects  6.1 Demonstration of the behavior | Environmental  Restructuring  Training | **Physical Opportunity** | Env |
|  | - Patients value social support in being active [2]. - Breaking sedentary bouts in cancer patients, emphasizing this approach is promising but new [26]. | - CC advises caregiver how to break sedentary bouts and encourages to support the patient by joining them in being active. |  | 12.2 Restruct. social environment  3.1 Social support (unspecified)  3.2 Social Support (practical) | Enablement | **Social Opportunity** | Si |
| **Reducing Sedentary Bouts** | - Patients strive for activity and have positive and negative attitudes towards eHealth, - 87% of cancer survivors are satisfied with Fitbit in intervention [27]. | - CC asks patient about the feasibility of performing the behavior and tells the patient that he/she can successfully perform the behavior. - CC and SMILeApp praises patient’s performance if the step goal was reached. |  | 9.1 Credible source  15.1 Verbal persuasion capability  10.4 Social reward | Persuasion  Incentivisation  Environmental restructuring  Modelling  Enablement  Education" | **Automatic Motivation** | Em  Reinf |
|  | - Patients have the intention /motivation to be active but are hampered by physical condition [2]. - Interrupting sedentary bouts with light-intensity activity helps control adiposity and postprandial glycaemia [28]. - Reducing/breaking up prolonged sedentary time decreases CV risk in general population [26]. - Exercise intervention improves QoL and fatigue in patients with hematological malignancies [29]. | - CC reviews the step goal and discrepancies at each visit together with patients. - SMILeApp provides graphical feedback on behavior. - CC asks the patient about the preference regarding intervals of breaking sedentary bouts (3/30 or 6/60) and set a step goal of daily steps until the next visit (optimal >5000 outpatient/ >1500 inpatient). - Patient defines a personal goal and enters it in the SMILeApp. - Patient identifies barriers and develops action plans to overcome barriers. - CC emphasizes the feasibility of the recommended behavior and individual goal of steps. - CC prompts patient to plan behavior and activities while breaking sedentary bouts. - CC checks knowledge on the devices (step counter and App) in follow-up visit. - SMILeApp and SMILeCare provide an overview of the daily steps and gives feedback regarding the outcome of behavior (daily steps). | As a patient I want to set a goal of daily steps so that I am later on able to check whether I reached them.  As a patient I want to have a graphical overview of my actual steps in relation to my step goal so that I see my progress. | 1.6 Discrepancy behavior and goal  1.1 Goal setting (behavior)  1.3 Goal setting (outcome)  8.3 Habit formation  1.2 Problem solving  1.4 Action planning  1.5 Review behavior goals  1.7 Review outcome goal | Persuasion  Incentivisation | **Reflective Motivation** | Int  B cons  B cap  G |

*Note:* BCT=Behavior Change Technique; COM-B= Capability, Opportunity Motivation Behavior; TDF=Theoretical Domains Framework; domains: Knowledge=K; Skills=S; Memory attention, decision processes=MAD; Behavioral regulation=Br; Social influences=Si; Environmental context and resources= Env; Emotion=Em; Intension= Int; Beliefs about consequences= B cons; Beliefs about capabilities= B cap; Optimism= O; Goals= G; Social/professional role and identity= Id; Reinforcement=Reinf..

# References

1. Michie S, Atkins L, West R (2014) The behaviour change wheel: a guide to designing interventions. 2014. ISBN-13. Silverback Publishing, Surrey

2. Leppla L, Mielke J, Kunze M, Mauthner O, Teynor A, Valenta S, Vanhoof J, Dobbels F, Berben L, Zeiser R (2020) Clinicians and patients perspectives on follow-up care and eHealth support after allogeneic hematopoietic stem cell transplantation: A mixed-methods contextual analysis as part of the SMILe study. European Journal of Oncology Nursing 45:101723

3. Syrjala KL, Martin PJ, Lee SJ (2012) Delivering care to long-term adult survivors of hematopoietic cell transplantation. Journal of clinical oncology : official journal of the American Society of Clinical Oncology 30 (30):3746-3751. doi:10.1200/jco.2012.42.3038

4. Coolbrandt A, Van den Heede K, Vanhove E, De Bom A, Milisen K, Wildiers H (2011) Immediate versus delayed self-reporting of symptoms and side effects during chemotherapy: Does timing matter? European Journal of Oncology Nursing 15 (2):130-136

5. Basch E, Deal AM, Kris MG, Scher HI, Hudis CA, Sabbatini P, Rogak L, Bennett AV, Dueck AC, Atkinson TM (2015) Symptom monitoring with patient-reported outcomes during routine cancer treatment: a randomized controlled trial. Journal of Clinical Oncology:JCO630830

6. Basch E, Deal AM, Dueck AC, Scher HI, Kris MG, Hudis C, Schrag D (2017) Overall Survival Results of a Trial Assessing Patient-Reported Outcomes for Symptom Monitoring During Routine Cancer Treatment. JAMA

7. Warrington L, Absolom K, Conner M, Kellar I, Clayton B, Ayres M, Velikova G (2019) Electronic systems for patients to report and manage side effects of cancer treatment: systematic review. Journal of medical Internet research 21 (1):e10875

8. Taylor S, Bellhouse S, Allsop M, Radford J, Yorke J (2020) The Role of e-Health in the Delivery of Care for Patients with Hematological Cancers: A Systematic Literature Review. Telemedicine journal and e-health : the official journal of the American Telemedicine Association. doi:10.1089/tmj.2019.0231

9. Berger DP, Engelhardt R, Mertelsmann R (2010) Das rote Buch: Hämatologie und internistische Onkologie;[mit 145 Therapie-Protokollen]. Hüthig Jehle Rehm,

10. Boyce JM, Allegranzi B, Pittet D (2017) Barriers to Compliance. Hand Hygiene: A Handbook for Medical Professionals:85-88

11. Srigley JA, Furness CD, Gardam M (2016) Interventions to improve patient hand hygiene: a systematic review. Journal of Hospital Infection 94 (1):23-29

12. Chemaly RF, Shah DP, Boeckh MJ (2014) Management of respiratory viral infections in hematopoietic cell transplant recipients and patients with hematologic malignancies. Clinical infectious diseases 59 (suppl_5):S344-S351

13. Lin SY, Fetzer SJ, Lee PC, Chen CH (2011) Predicting adherence to health care recommendations using health promotion behaviours in kidney transplant recipients within 1–5 years post‐transplant. Journal of clinical nursing 20 (23‐24):3313-3321

14. Chen G, Kendall PA, Hillers VN, Medeiros LC (2010) Qualitative studies of the food safety knowledge and perceptions of transplant patients. Journal of food protection 73 (2):327-335

15. Lipkin AC, Lenssen P, Dickson BJ (2005) Nutrition issues in hematopoietic stem cell transplantation: state of the art. Nutrition in clinical practice 20 (4):423-439

16. Larsen J, Nordstrom G, Ljungman P, Gardulf A (2004) Symptom occurrence, symptom intensity, and symptom distress in patients undergoing high-dose chemotherapy with stem-cell transplantation. Cancer nursing 27 (1):55-64

17. Rebafka A (2016) Medication Adherence After Renal Transplantation—a Review of the Literature. Journal of renal care 42 (4):239-256

18. Jamieson NJ, Hanson CS, Josephson MA, Gordon EJ, Craig JC, Halleck F, Budde K, Tong A (2016) Motivations, challenges, and attitudes to self-management in kidney transplant recipients: a systematic review of qualitative studies. American Journal of Kidney Diseases 67 (3):461-478

19. Conn VS, Ruppar TM (2017) Medication adherence outcomes of 771 intervention trials: systematic review and meta-analysis. Preventive medicine 99:269-276

20. Tong A, Howell M, Wong G, Webster AC, Howard K, Craig JC (2011) The perspectives of kidney transplant recipients on medicine taking: a systematic review of qualitative studies. Nephrology Dialysis Transplantation 26 (1):344-354

21. Low JK, Crawford K, Manias E, Williams A (2016) A compilation of consumers’ stories: the development of a video to enhance medication adherence in newly transplanted kidney recipients. Journal of advanced nursing 72 (4):813-824

22. Cooke L, Chung C, Grant M (2011) Psychosocial care for adolescent and young adult hematopoietic cell transplant patients. Journal of psychosocial oncology 29 (4):394-414

23. Williams A, Low JK, Manias E, Crawford K (2016) The transplant team's support of kidney transplant recipients to take their prescribed medications: a collective responsibility. Journal of clinical nursing 25 (15-16):2251-2261

24. Morishita S, Kaida K, Yamauchi S, Wakasugi T, Ikegame K, Ogawa H, Domen K (2017) Relationship of physical activity with physical function and health‐related quality of life in patients having undergone allogeneic haematopoietic stem‐cell transplantation. European journal of cancer care 26 (4):e12669

25. Bevans M, El-Jawahri A, Tierney DK, Wiener L, Wood WA, Hoodin F, Kent EE, Jacobsen PB, Lee SJ, Hsieh MM (2017) National Institutes of Health Hematopoietic Cell Transplantation Late Effects Initiative: The Patient-Centered Outcomes Working Group Report. Biology of Blood and Marrow Transplantation 23 (4):538-551

26. Howden EJ, La Gerche A, Arthur JF, McMullen JR, Jennings GL, Dunstan DW, Owen N, Avery S, Kingwell BA (2018) Standing up to the cardiometabolic consequences of hematological cancers. Blood reviews 32 (5):349-360

27. Gell NM, Grover KW, Humble M, Sexton M, Dittus K (2017) Efficacy, feasibility, and acceptability of a novel technology-based intervention to support physical activity in cancer survivors. Supportive Care in Cancer 25 (4):1291-1300

28. Chastin SF, Egerton T, Leask C, Stamatakis E (2015) Meta‐analysis of the relationship between breaks in sedentary behavior and cardiometabolic health. Obesity 23 (9):1800-1810

29. Knips L, Bergenthal N, Streckmann F, Monsef I, Elter T, Skoetz N (2019) Aerobic physical exercise for adult patients with haematological malignancies. Cochrane Database of Systematic Reviews (1)
